# Myofibrillar myopathy: a rare but important differential diagnosis of camptocormia in a patient with Parkinson’s Disease

**DOI:** 10.1186/s42466-023-00250-y

**Published:** 2023-06-08

**Authors:** Jan Niklas Petry-Schmelzer, Angela Abicht, Michael T. Barbe, Gilbert Wunderlich

**Affiliations:** 1 Department of Neurology, Faculty of Medicine and University Hospital, University of Cologne, Kerpener Straße 62, 50937 Cologne, Germany; 2 Center for Rare Diseases, Faculty of Medicine and University Hospital, University of Cologne, Cologne, Germany; 3 Medizinisch Genetisches Zentrum (MGZ) München, Munich, Germany; 4 Department of Neurology, Friedrich-Baur-Institute, Ludwig-Maximilians-University Munich, Munich, Germany

**Keywords:** Axial myopathy, Tetraparesis

## Abstract

Here we report on a patient with Parkinson's Disease and camptocormia due to Myofibrillar Myopathy Type 3. By leading the reader through the clinical reasoning process and highlighting the respective red flags we aim to increase the readers’ awareness for the differential diagnosis of camptocormia.

**Supplementary Information:**

The online version contains supplementary material available at 10.1186/s42466-023-00250-y.

Camptocormia represents one of the hallmark motor symptoms in advanced Parkinson’s Disease, occurring in 3 to 18% of the patients [1, 2]. Although being crucial for prognosis and further treatment, it might be challenging to correctly determine its etiology, especially when there are several concurring causes of camptocormia present.

A 78-year-old female patient presented with a six-year history of progressive bending-forward during walking and weakness of the legs, reported as occasional tendency to fall because of giving-away of the legs. Therefore, five years prior to admission, the patient underwent decompression of a lumbar spinal stenosis, which was initially suspected as the cause of change in posture. Two years later, an additional resting tremor of the right hand and micrography accompanied by hyposmia and constipation were recognized. Family history was unremarkable for neurologic diseases. Medical history comprised arterial hypertension, surgically treated bilateral cataract, and a ruptured tendon of the right m. supraspinatus. Cerebral MRI was unremarkable and dopamine transporter scintigraphy showed a bilateral striatal dopaminergic deficit in line with the described Parkinsonian syndrome. Dopaminergic treatment was initiated, resulting in alleviation of resting tremor and micrography. However, neither spinal decompression nor dopaminergic treatment led to an improvement of posture during gait or weakness of the legs.

On neurological examination (see Additional file 1: Video 1) there was hypomimia, dysarthrophonia, rigidity and hypokinesia pronounced on the right without any tremor under dopaminergic treatment. Tendon reflexes were attenuated on the lower extremities and normal on the upper extremities. There were no pyramidal signs. Sensation and coordination were unremarkable. The patient was not able to raise from a chair without using her arms and a moderate tetraparesis with axial involvement was obvious. When standing straight there was an anterior trunk flexion (estimated lumbar fulcrum 15–20°) with worsening to camptocormia while walking (estimated lumbar fulcrum > 30°). The patient had Trendelenburg gait with normal stride length and without festination.

MRI of the spine showed fatty atrophy of the paraspinal muscles without contrast enhancement. Electromyography revealed pathologic spontaneous activity in the m. biceps brachii, m. interosseus dorsalis manus I, m. tibialis anterior and the lumbar and thoracic paraspinal muscles, accompanied by myotonic discharges in all examined muscles except the m. biceps brachii. Myopathically altered muscle unit potentials were found especially in the paraspinal muscles and the m. biceps brachii. Laboratory testing including creatine kinase (CK) was normal. Genetic testing for myotonic and myopathic disorders revealed a heterozygous pathogenic variant in the *MYOT* gene (c.179C>T p.(Ser60Phe)), leading to the diagnosis of an autosomal dominant myofibrillary myopathy type 3 as the cause of camptocormia [3].

This case aims to highlight the importance of a proper anamnesis and dedicated neurological examination in patients with camptocormia, although if the suspected cause of camptocormia seems obvious at first sight. This is of especial importance if one is faced the challenge of several concurring causes of camptocormia [2]. Among neurodegenerative diseases, accounting for approximately 40% of camptocormia, camptocormia occurs most often in Parkinson’s Disease. Although the exact pathogenesis of camptocormia in PD is not clearly understood [2, 4], it only very rarely occurs prior to any cardinal motor symptoms [5], and especially the report of early weakness of the legs pointed towards an underlying neuromuscular disorder. Neuromuscular and musculoskeletal disorders can be found in approx. 50% of patients with camptocormia, while immune-mediated and drug-induced camptocormia is either rare (approx. 5%) [1, 6]. Therefore, primary muscular diseases with axial involvement, focal myositis and secondary myopathies associated with neurodegenerative disease tend to be the most frequent causes of camptocormia. Myofibrillar myopathy, as present in this case, is characterized by disintegration of myofibrils predominantly at the Z-disc level and sarcoplasmic protein aggregates, leading to distal and proximal weakness, sometimes combined with axial weakness, and in a minority of patients, cardiomyopathy, and respiratory insufficiency [7]. There are several gene mutations known to cause myofibrillar myopathy which most likely arise in infancy or early to middle adulthood. However, the onset of the presented autosomal-dominant variant in the *MYOT* gene is in middle to late adulthood, highlighting its importance as differential diagnosis of axial myopathy in the elderly patient [8]. A normal level of CK is not uncommon in myofibrillar myopathy, although there are also reports of a two-fold increased CK [3]. Fatty atrophy of the spinal muscles in MRI has also been described in camptocormia caused by Parkinson’s Disease and therefore cannot solely indicate an underlying myopathy [1].

To conclude and as demonstrated by the present case, in patients with Parkinson’s Disease and camptocormia special attention should be paid to medical history (here: onset of camptocormia prior to onset of other motor symptoms) and neurological examination (here: moderate tetraparesis). If suspicious, additional diagnostic procedures including MRI of the spine, EMG, laboratory testing and eventually muscle biopsy or genetic testing is recommended to rule out potentially treatable causes and to follow-up on the patient properly (here: monitoring for pulmonary and cardiac function).

### Supplementary Information

**Additional file 1.** Video 1: Neurological examination of the patient shows an akinetic rigid syndrome pronounced on the right with camptocormia and moderate tetraparesis.

## Data Availability

The datasets used during the current study are available from the corresponding author on reasonable request.

## Abbreviations

CK: Creatine kinase
EMG: Electromyography
MRI: Magnetic resonance imaging
